# Bifurcation analysis of a two-compartment hippocampal pyramidal cell model

**DOI:** 10.1007/s10827-016-0606-8

**Published:** 2016-05-25

**Authors:** Laura A. Atherton, Luke Y. Prince, Krasimira Tsaneva-Atanasova

**Affiliations:** Engineering Mathematics, and Physiology, Pharmacology & Neuroscience, University of Bristol, Bristol, England UK; Physiology, Pharmacology & Neuroscience, University of Bristol, Bristol, England UK; Department of Mathematics, College of Engineering, Mathematics and Physical Sciences, & EPSRC Centre for Predictive Modelling in Healthcare, University of Exeter, Exeter, Devon EX4 4QF UK

**Keywords:** Dynamical system, Bifurcation analysis, Bursting and spiking, Numerical continuation, Parameter dependence

## Abstract

The Pinsky-Rinzel model is a non-smooth 2-compartmental CA3 pyramidal cell model that has been used widely within the field of neuroscience. Here we propose a modified (smooth) system that captures the qualitative behaviour of the original model, while allowing the use of available, numerical continuation methods to perform full-system bifurcation and fast-slow analysis. We study the bifurcation structure of the full system as a function of the applied current and the maximal calcium conductance. We identify the bifurcations that shape the transitions between resting, bursting and spiking behaviours, and which lead to the disappearance of bursting when the calcium conductance is reduced. Insights gained from this analysis, are then used to firstly illustrate how the irregular spiking activity found between bursting and stable spiking states, can be influenced by phase differences in the calcium and dendritic voltage, which lead to corresponding changes in the calcium-sensitive potassium current. Furthermore, we use fast-slow analysis to investigate the mechanisms of bursting and show that bursting in the model is dependent on the intermediately slow variable, calcium, while the other slow variable, the activation gate of the afterhyperpolarisation current, does not contribute to setting the intraburst dynamics but participates in setting the interburst interval. Finally, we discuss how some of the described bifurcations affect spiking behaviour, during sharp-wave ripples, in a larger network of Pinsky-Rinzel cells.

## Introduction

An extensive body of literature has implicated the hippocampal formation in spatial navigation and episodic memory (O’Keefe and Nadal 1978; Burgess et al. 2002; Morris et al. 1982; Riedel et al. 1999; Squire 1992). Within the hippocampus, the recurrent connectivity within the autoassociative CA3 network on the one hand confers onto the region a strong computational capacity which can mediate behavioural processes such as pattern completion (McNaughton and Morris 1987; Rolls and Kesner 2006; Nakazawa et al. 2002) while on the other hand predisposes the region to epileptiform activity (Traub and Wong 1982; Miles and Wong 1983; Le Duigou et al. 2014). At the cellular level, CA3 pyramidal cells intrinsically display a variety of firing patterns, ranging from single action potentials to complex bursts (Kandel and Spencer 1961; Traub et al. 1991; Mizuseki et al. 2012; Wong and Prince 1978; 1981; Spruston and McBain 2007). Such bursting is important for place cell activity (Harvey et al. 2009; Epsztein et al. 2011; Bittner et al. 2015), signal propagation and the induction of synaptic plasticity (Lisman 1997; Buchanan and Mellor 2010).

In an attempt to understand the behaviour of isolated cells and the coupled CA3 network, numerous computational models of CA3 pyramidal cells have been proposed, ranging from detailed, multi-compartmental models down to single compartmental models (Traub et al. 1991; Pinsky and Rinzel 1994; Migliore et al. 1995; Grobler et al. 1998; Xu and Clancy 2008; Nowacki et al. 2011). The Pinsky-Rinzel model (Pinsky and Rinzel 1994) was originally formulated over 20 years ago as a two-compartment reduction of the 19-compartment Traub CA3 cell model, developed earlier (Traub et al. 1991). Since then variants of Pinsky-Rinzel model have been used to investigate hippocampal sharp wave ripple oscillations (Taxidis et al. 2012); carbachol-induced gamma oscillations (Tiesinga et al. 2001); rate and temporal coding of place cells (Kamondi et al. 1998; Booth and Bose 2001); and the influence of dendritic morphology on firing patterns (Mainen and Sejnowski 1996), to name but a few examples.

Whilst parameter regimes for tonic spiking and bursting activity were investigated in the original Pinsky-Rinzel paper (Pinsky and Rinzel 1994), a detailed mathematical analysis of the dynamical regimes of the model has only ever been performed once (Hahn and Durand 2001). This is likely because the Pinsky-Rinzel model is non-smooth, and so traditional techniques used to study dynamical systems cannot be employed. In this previous paper, bifurcation analysis (Strogatz 2001) was used to investigate the transitions between resting, bursting and spiking states as the size of the extracellular potassium concentration was increased (Hahn and Durand 2001). However, a detailed description of how this analysis was performed, given the non-smooth nature of the system, was never provided. Due to the complexity of the model, a great deal of extra insight can be gained by analysing how some of the many other parameters shape the dynamical landscape of the model. This can then inform parameter choices and potentially explain dynamic behaviour in larger networks of Pinsky-Rinzel cells, such as Taxidis et al. (2012), which are much more difficult to analyse.

Therefore we recast the original model equations using fully continuous functions. This permitted the use of available numerical continuation methods to perform bifurcation analysis using three notable bifurcation parameters: the applied somatic and dendritic currents, and the maximum calcium conductance. These first two parameters were chosen in order to ask what the mathematical mechanisms were for the originally observed transitions between resting, bursting and spiking as the applied current was increased (Pinsky and Rinzel 1994). This is of particular interest for Pinsky-Rinzel cell behaviour in a larger network, where excitatory and inhibitory inputs may impinge onto either the somatic or dendritic compartment, or both. Would the mechanisms behind the transitions be qualitatively similar from current applied to either compartment? Or would the mechanisms differ? Having understood these transitions, the third bifurcation parameter was chosen because lowering the maximum calcium conductance can switch behaviour from bursting to spiking (Pinsky and Rinzel 1994; Traub et al. 1991), as has been used to better represent the firing properties of CA1 cells (Taxidis et al. 2012). How does this occur from dynamical systems point of view? Is it just the bursting that disappears, or do other behaviours also change as the calcium conductance is reduced, and if so how might calcium be involved in shaping some of these behaviours? As an example of how answers to the above questions could be used in informing understanding of network activity, we then discuss the implications for some of the bifurcations identified, in relation to a larger network of Pinsky-Rinzel cells, exhibiting sharp-wave ripple oscillations (Taxidis et al. 2012).

Finally, given the relationship between the maximum calcium conductance and bursting, fast slow analysis (Rinzel 1987) was used to further investigate the intraburst dynamics. Traditionally this has been used to isolate computationally the important variables, responsible for bursting, given the difficulty of teasing apart the system experimentally. When this technique was applied to two separate pyramidal cell models based on, but slightly different to, the Pinsky-Rinzel model; bursting behaviour was either of the square-wave/fold-homoclinic type and dependent on the activation variable of the slow potassium current (Kepecs and Wang 2000) or of the parabolic type and dependent on both the slow autocatalytic activation variable for a T-type calcium channel and the activation variable for the slow calcium-dependent potassium current (Xu and Clancy 2008). Given these differences, it is difficult to interpolate which mechanism of bursting might exist in the original model, for which fast-slow analysis has never been performed. Moreover, while in the original paper, the authors describe burst initiation as occurring when the slow activation variable, (*q*), for the potassium afterhyperpolarisation current fell below a threshold value (Pinsky and Rinzel 1994); subsequent studies have shown instead, using phase-plane analysis on a piecewise reduced system of the original model, that although *q* is important for controlling the interburst interval, it is not important for setting the burst initiation threshold (Booth and Bose 2001; Bose and Booth 2005). Therefore we perform fast-slow analysis, using the modified continuous version of the Pinsky-Rinzel model we propose here, in an attempt to clarify the role of *q* and also investigate its interaction with the other slow variable, calcium (*Ca*), in controlling the burst dynamics.

## Model

The Pinsky-Rinzel model characterises a typical pyramidal cell as comprising a single axosomatic and a single dendritic compartment, where the somatic compartment contains a transient sodium *I*_Na_, delayed rectifier potassium $I_{\mathrm {K_{\text {DR}}}}$, and leak current. The dendritic compartment contains a persistent calcium *I*_Ca_, calcium activated potassium $I_{\mathrm {K_{\text {Ca}}}}$, after-hyperpolarisation potassium current $I_{\mathrm {K_{\text {AHP}}}}$, and leak current. The two compartments are coupled by a coupling current *I*_SD_ or *I*_DS_ and their membrane voltages summarised by the following differential equations:

$$\begin{array}{@{}rcl@{}} C_{\mathrm{m}} \frac{dV_{\mathrm{s}}}{dt} &=& -I_{\text{Leak}} - I_{\text{Na}} - I_{\mathrm{K_{\text{DR}}}} - \frac{I_{\text{DS}}}{p} + \frac{I_{\mathrm{S_{\text{app}}}}}{p} \\ C_{\mathrm{m}} \frac{dV_{\mathrm{d}}}{dt} &=& -I_{\text{Leak}} - I_{\text{Ca}} - I_{\mathrm{K_{\text{Ca}}}} - I_{\mathrm{K_{\text{AHP}}}} + \\ &&+ \frac{I_{\text{SD}}}{(1-p)} +\frac{ I_{\mathrm{D_{\text{app}}}}}{(1-p)}, \end{array} $$

that evolve in time (measured in milliseconds).

All currents are conductance-based, using the Hodgkin-Huxley formalism (Hodgkin and Huxley 1952) of activation and inactivation gates *m*,*h*,*n*,*s*,*c*,*q* dependent on voltage or intracellular calcium that drive the current. Additionally, $I_{\mathrm {K_{\text {Ca}}}}$ has a saturation function dependent on calcium *χ*(*Ca*).

$$\begin{array}{@{}rcl@{}} I_{\text{Na}} &=& g_{\text{Na}}\:m_{\infty}^{2}(V_{\mathrm{s}})\:h\:(V_{\mathrm{s}}-V_{\text{Na}}) \\ I_{\mathrm{K_{\text{DR}}}} &=& g_{\mathrm{K_{\text{DR}}}}\:n\:(V_{\mathrm{s}}-V_{\mathrm{K}}) \\ I_{\text{Ca}} &=& g_{\text{Ca}}\:s^{2}\:(V_{\mathrm{d}} - V_{\text{Ca}}) \\ I_{\mathrm{K_{\text{Ca}}}}&=&g_{\mathrm{K_{\text{Ca}}}}\:c\:\chi(Ca)\:(V_{\mathrm{d}}-V_{\mathrm{K}})\\ I_{\mathrm{K_{\text{AHP}}}}&=& g_{\mathrm{K_{\text{AHP}}}}\:q\:(V_{\mathrm{d}} - V_{\mathrm{K}}) \\ I_{\text{SD}} &=& -I_{\text{DS}} = g_{\mathrm{C}}\:(V_{\mathrm{d}} - V_{\mathrm{s}}) \\ I_{\text{Leak}} &=& g_{\mathrm{L}}\:(V - V_{\mathrm{L}}) \end{array} $$

Maximal conductance parameters were taken (in *μ* S/cm ^2^) as *g*_Na_=30, $g_{\mathrm {K_{\text {DR}}}}=15$, $g_{\mathrm {K_{\text {Ca}}}}=15$, $g_{\mathrm {K_{\text {AHP}}}}=0.8$, *g*_Ca_=10, *g*_L_=0.1 and *g*_C_=2.1, while reversal potentials were taken (in mV) as *V*_Na_=60, *V*_K_=−75, *V*_Ca_=80, and *V*_L_=−60. The size of the axosomatic compartment as a proportion of the entire cell was given by *p*=0.5 and that of the dendritic compartment as 1−*p*. The activation and inactivation gates evolve as a function of their steady state activation *x*_*∞*_, and time constant *τ*_*x*_ curves, where *U* represents the membrane potentials *V*_s_ or *V*_d_, or the intracellular calcium *Ca*.

$$\frac{dx}{dt} = \frac{x_{\infty}(U) - x}{\tau_{x}(U)} $$

*x*_*∞*_ and *τ*_*x*_ are often expressed in terms of forward and backward rate functions *α* and *β*.

$$\begin{array}{@{}rcl@{}} x_{\infty}(U) &=& \frac{\alpha_{x}(U)}{\alpha_{x}(U)+\beta_{x}(U)} \\ \tau_{x}(U) &=& \frac{1}{\alpha_{x}(U)+\beta_{x}(U)}. \end{array} $$

In the original formulation, the *m*, *h*, *n*, and *s* variables are driven solely by continuous rate functions, whereas the *c*, *q*, and *χ* are given as discontinuous rate functions, where *H*() is the Heaviside step function,

$$\begin{array}{@{}rcl@{}} \alpha_{m}(V_{\mathrm{s}}) &=& \frac{0.32(-46.9-V_{\mathrm{s}})}{(\exp((-46.9-V_{\mathrm{s}})/4)-1)} \\ \beta_{m}(V_{\mathrm{s}}) &=& \frac{0.28(V_{\mathrm{s}}+19.9)}{(\exp((V_{\mathrm{s}}+19.9)/5)-1)} \\ \alpha_{n}(V_{\mathrm{s}}) &=& \frac{0.016(-24.9-V_{\mathrm{s}})}{(\exp((-24.9-V_{\mathrm{s}})/5)-1)} \\ \beta_{n}(V_{\mathrm{s}}) &=& 0.25\exp(-1-0.025V_{\mathrm{s}}) \\ \alpha_{h}(V_{\mathrm{s}}) &=& 0.128\exp(\frac{(-43-V_{\mathrm{s}})}{18}) \\ \beta_{h}(V_{\mathrm{s}}) &=& \frac{4}{(1+\exp((-20-V_{\mathrm{s}})/5))} \\ \alpha_{s}(V_{\mathrm{d}}) &=& \frac{1.6}{(1+\exp(-0.072(V_{\mathrm{d}}-5)))} \\ \beta_{s}(V_{\mathrm{d}}) &=& \frac{0.02(V_{\mathrm{d}}+8.9)}{(\exp((V_{\mathrm{d}}+8.9)/5)-1)} \\ \alpha_{c}(V_{\mathrm{d}}) &=& \frac{(1-H(V_{\mathrm{d}}+10))\exp((V_{\mathrm{d}}+50)/11-(V_{\mathrm{d}}+53.5)/27)}{18.975} \\ &&+H(V_{\mathrm{d}}+10)\:(2\exp(\frac{(-53.5-V_{\mathrm{d}})}{27})) \\ \beta_{c}(V_{\mathrm{d}}) &=& (1-H(V_{\mathrm{d}}+10))(2\exp(\frac{(-53.5-V_{\mathrm{d}})}{27})-\alpha_{c}(V_{\mathrm{d}})) \\ \alpha_{q}(Ca) &=& \min(0.00002\:Ca,0.01) \\ \beta_{q}(Ca) &=& 0.001 \\ \chi(Ca) &=& \min(\frac{Ca}{250},1) \end{array} $$

**Fig. 1:**
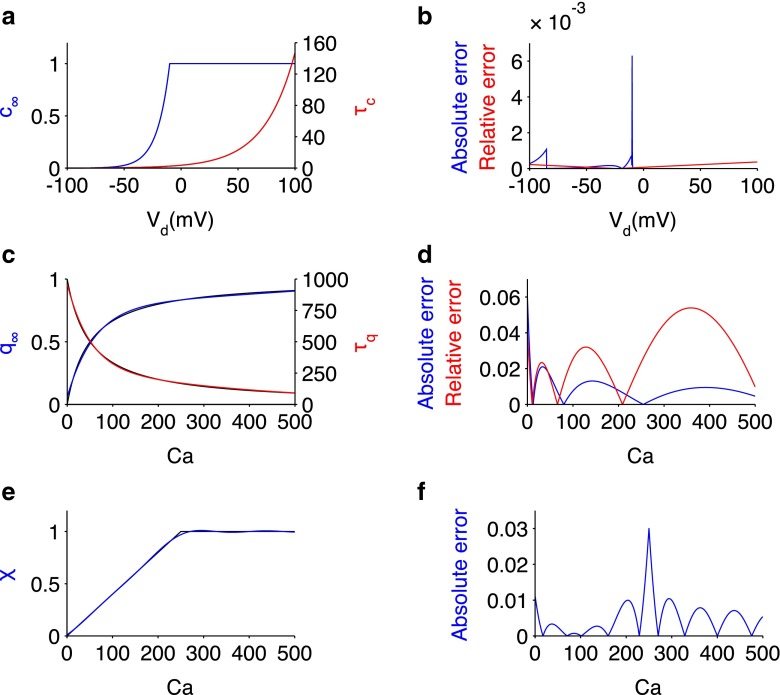
Approximation of discontinuous function. Approximated functions (**a**, **c**, **e**) and corresponding error of approximation (**b**, **d**, **f**) for $c_{\infty }$, (*blue*) and *τ*_*c*_ (*red*) (A-B), $q_{\infty }$ (*blue*) and *τ*_*q*_ (*red*) (**c**–**d**), and *χ* (*blue*) (**e**). For the approximated functions, the original discontinuous functions are plotted in *black*. Relative error was used for the time constant functions (*τ*_*c*_ and *τ*_*q*_), where error was computed over a large range of values, while absolute error was used for $c_{\infty }$, $q_{\infty }$ and *χ*

**Fig. 2:**
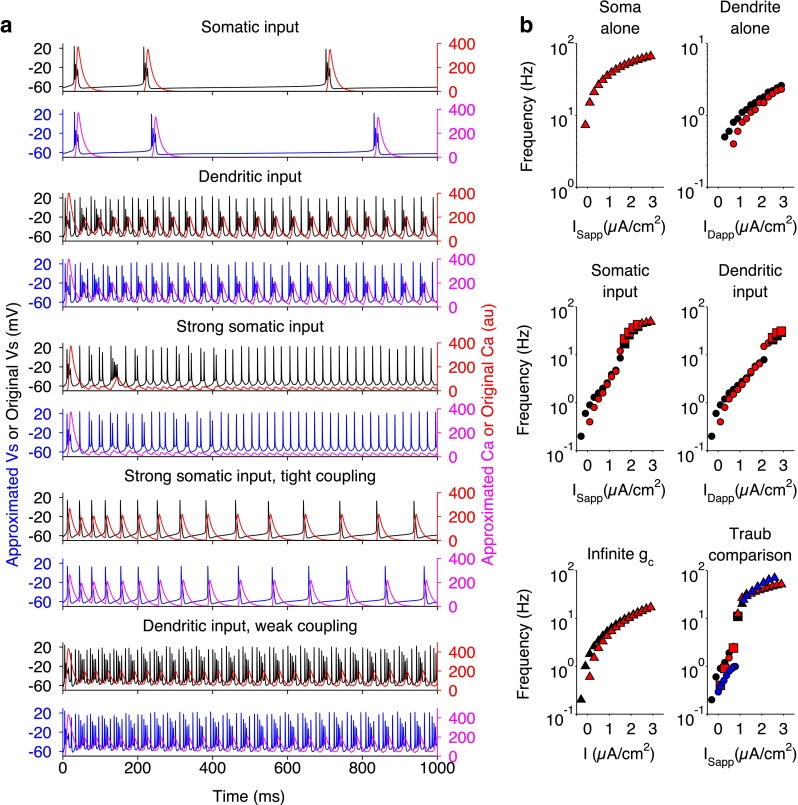
Comparison of firing patterns and f/I curves of the original model and our approximated model. **a** *V*_s_ and *C* *a* in the original (*black* and *red*, respectively) and approximated (*blue* and *magenta*, respectively) models for different parameter regimes as follows: somatic input (*I*_Sapp_=0.75), dendritic input (*I*_Dapp_=−0.5, *g*_NMDA_=1.25), strong somatic input (*I*_Sapp_=2.5), somatic input with tight coupling (*I*_Sapp_=2.5, *g*_C_=10.5), and dendritic input with weak coupling (*I*_Dapp_=−0.5, *g*_NMDA_=1.25, *g*_C_=1.425). **b** f/I curves in the original model (*black*) or approximated model (*red*) for the isolated soma or dendrite (*upper panels*) and the 2-compartment model with standard *g*_C_=2.1 (*middle panels*), infinite *g*_C_ (*lower left panel*) and *g*_C_=1.85 (*lower right panel*) for comparison with the Traub model (Traub et al. 1991) in *blue*. *Circles* indicate burst frequency, *triangles* are spike frequency and *squares* are mixed burst-spike frequency

To allow us to perform bifurcation analysis, we approximated the discontinuous functions by fitting continuous functions directly to the steady state and time activation curves. A comparison of the original and fitted curves are shown in Fig. 1, which shows a small relative error for each fitted curve, of the order 10^−2^−10^−3^. To confirm that the approximated system displayed the same behaviour as the original system, we reproduced the firing patterns and f/I curve of the original model (Fig. 2). The approximated functions are as follows,

$$\begin{array}{@{}rcl@{}} c_{\infty}(V_{\mathrm{d}}) &=&(1/(1+\exp((-10.1-V_{\mathrm{d}})/0.1016)))^{0.00925},\\ \tau_{c}(V_{\mathrm{d}}) &=&3.627\exp(0.03704\:V_{\mathrm{d}}), \\ q_{\infty}(Ca) &=& (0.7894\exp(0.0002726\:Ca))\\ &&-(0.7292\exp(-0.01672\:Ca)), \\ \tau_{q}(Ca) &=& (657.9\exp(-0.02023\:Ca))\\ &&+(301.8\exp(-0.002381\:Ca)),\\ \chi(Ca) &=& 1.073\sin(0.003453\:Ca+0.08095)\\ &&+0.08408\sin(0.01634\:Ca-2.34)\\&&+0.01811\sin(0.0348\:Ca-0.9918). \end{array} $$

Intracellular free calcium in the effective sub-membrane shell is given as dimensionless, since in the original model it was reasoned that the size of the effective shell was undetermined, so appropriate scaling could not be given. Nevertheless, this evolves according to

$$\begin{array}{@{}rcl@{}} \frac{dCa}{dt} = -0.13 I_{Ca} - 0.075 Ca. \end{array} $$

Bifurcation and continuation analysis was conducted in XPPAUT, a tool for simulating and analysing dynamical systems (Ermentrout 2002). One- and two-parameter bifurcation diagrams were constructed using AUTO within XPPAUT. For fast-slow analysis, the differential equations describing *Ca* and *q* were independently removed from the full system of equations and instead *Ca* and *q* were treated as parameters. All Figures were constructed in MATLAB. The code for the model simulations is available on ModelDB and could be found here: https://senselab.med.yale.edu/ModelDB/ShowModel.cshtml?model=189088.

## Results

### Response to steady somatic applied current

In the original paper, as the applied current to the somatic or dendritic compartment was increased, the authors saw various qualitative shifts in behaviour between resting, bursting and spiking (Pinsky and Rinzel 1994). Understanding the dynamic mechanisms behind these transitions is important because, within a neural network, individual Pinsky-Rinzel cells do not operate in isolation but rather there are various factors which might affect the net current impinging onto the somatic and dendritic compartments, most notably excitatory and inhibitory synaptic input and also the presence of neuromodulatory factors. Therefore we begin by investigating the effect of somatically or dendritically applied current on the dynamical behaviour of the cell using bifurcation analysis.

**Fig. 3:**
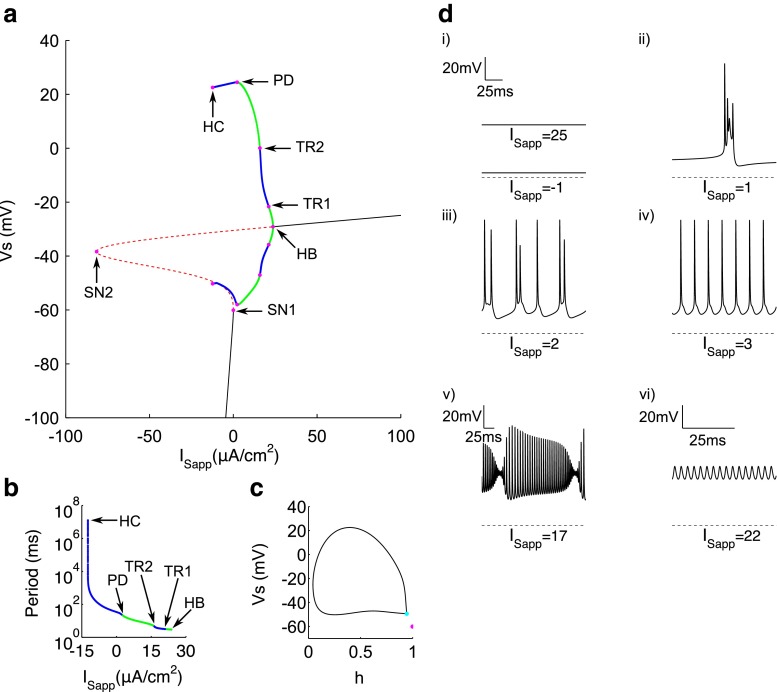
Bifurcation analysis for applied somatic current. **a** Bifurcation diagram with *I*_Sapp_ as the bifurcation parameter. Stable nodes (*black line*), saddles (*red dashed line*), stable periodic orbit maxima and minima (*green dots*), unstable periodic orbit maxima and minima (*blue dots*), bifurcation points (*magenta dots*). SN1 saddle node bifurcation 1, SN2 saddle node bifurcation 2, HB supercritical hopf bifurcation, TR1 torus bifurcation 1, TR2 torus bifurcation 2, PD period doubling bifurcation, HC homoclinic bifurcation. **b** Period of limit cycle solutions. **c** Phase portrait in *V*_s_ and *h* at the homoclinic bifurcation point. SN1 (*magenta*), saddle at homoclinic bifurcation (*cyan dot*). D) Representative traces of somatic voltage at different values of *I*_Sapp_. *Dashed line* represents −75 mV for reference

The bifurcation diagram using *I*_Sapp_ as the initial bifurcation parameter is formed of an S-shaped curve of steady states and a curve of periodic orbits (Fig. 3a). The lower branch of the S-curve consists of stable nodes (see Fig. 3di, *I*_Sapp_=−1) which correspond to the hyperpolarised resting state of the cell. The system is driven to these nodes predominantly by the potassium-based *I*_Leak_, in accordance with experimental work (Bernstein 1902; Hodgkin and Horowicz 1959). Stability is lost via a saddle-node bifurcation (SN1) at *I*_Sapp_=0.02651, which represents the rheobase for *I*_Sapp_ and gives rise to a branch of saddles, which forms the middle and part of the upper branches of the S-curve. This branch of saddles turns around at another saddle-node bifurcation (SN2) at *I*_Sapp_=−81.57 before regaining stability at the supercritical Hopf bifurcation (HB) for *I*_Sapp_=23.69. For increasing *I*_Sapp_ there remains a branch of stable nodes, corresponding to a depolarised resting state of around -30mV (see Fig. 3di, *I*_Sapp_=25).

A branch of stable periodic solutions emerges from the HB with low amplitude and high frequency (see Fig. 3dvi, *I*_Sapp_=22). These solutions are only apparent in a small range of *I*_Sapp_ since stability is lost for decreasing *I*_Sapp_ between two torus bifurcations (TR1 at *I*_Sapp_=21.14 and TR2 at *I*_Sapp_=15.87). Within this range, an undulating spiking pattern is observed with increasing and then decreasing spike amplitude (see Fig. 3dv, *I*_Sapp_=17). For *I*_Sapp_ below that at TR2, a second branch of stable periodic solutions emerges, representing regular spiking activity (see Fig. 3div, *I*_Sapp_=3) with greater spike amplitude but lower frequency than the first branch of stable spiking solutions (Fig. 3b), as described in the original model (Pinsky and Rinzel 1994). Such spiking is dominated by the dynamics of *I*_Na_ and *I*_K_ in the somatic compartment, as well described by seminal work by Hodgkin and Huxley (1952). The aperiodic and spike doubling behaviour for lower *I*_Sapp_ (see Fig. 3diii , *I*_Sapp_=2) is brought about via the period doubling (PD) bifurcation which occurs at *I*_Sapp_=2.288 and leads to a final branch of unstable periodic solutions. For further decreasing *I*_Sapp_, aperiodic and spike doubling behaviour transitions to very low frequency (VLF) bursting as in the original model (see Fig. 3dii). The currents involved in shaping these latter behaviours are discussed in more detail later in this paper. Finally, the periodic solutions disappear via a homoclinic bifurcation (HC) at *I*=−12.35 at which point the period is infinitely large (Fig. 3b). A homoclinic rather than saddle-node on infinite circle (SNIC) bifurcation was confirmed by showing that a very high period (1.27×10^7^) limit cycle joins together with a saddle for this value of *I*_Sapp_, rather than coalescing with the saddle at the SN1 bifurcation (Fig. 3c).

**Fig. 4:**
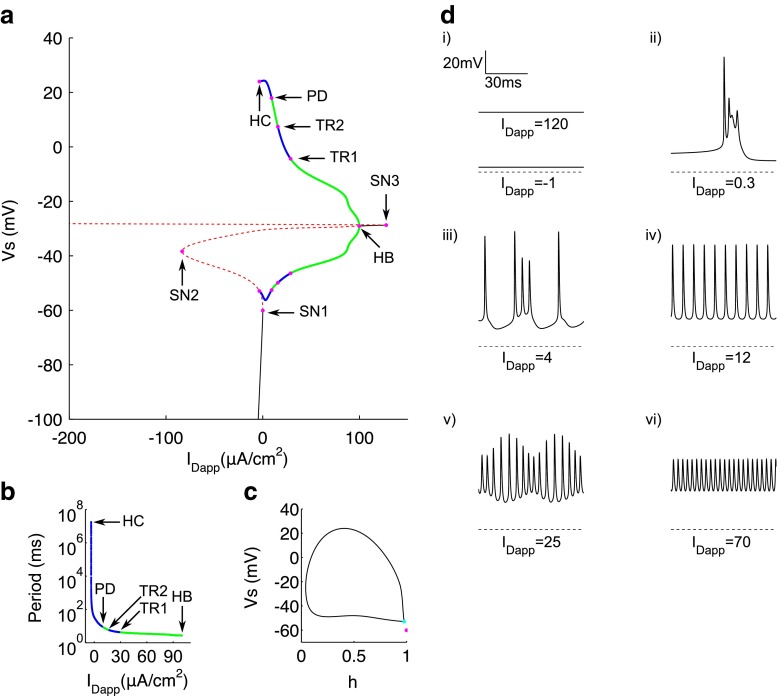
Bifurcation analysis for applied dendritic current. **a** Bifurcation diagram with *I*_Dapp_ as the bifurcation parameter. Stable nodes (*black line*), saddles (*red dashed line*), stable periodic orbit maxima and minima (*green dots*), unstable periodic orbit maxima and minima (*blue dots*), bifurcation points (*magenta dots*). SN1 saddle node bifurcation 1, SN2 saddle node bifurcation 2, HB supercritical hopf bifurcation, SN3 saddle node bifurcation 3, TR1 torus bifurcation 1, TR2 torus bifurcation 2, HC homoclinic bifurcation. **b** Period of limit cycle solutions. **c** Phase portrait in *V*_*s*_ and *h* at the homoclinic bifurcation point. SN1 represented by magenta dot and saddle at homoclinic bifurcation represented by cyan dot. **d** Example traces of somatic voltage at different values of *I*_Dapp_. *Dashed line* represents −75 mV for reference

It is important to note that for the numerical continuation results presented in Figs. 3a and 4a, we consider a very large range of the bifurcation parameter values, namely *I*_Sapp/Dapp_∈[−500,500], in order to locate the Hopf bifurcations giving rise to stable spiking solutions. The large range of the bifurcation parameter increases the likelihood of finding turning points for the curve of steady states. Interpreting the entirety of this range as meaningful should be cautioned. It is extremely unlikely that the currents required to reach the solutions between the Torus bifurcations and between the Torus and Hopf bifurcations in the model would ever be physiologically relevant, given that the unitary amplitude of a single CA3-CA3 monosynaptic EPSC is of the order of 20–30 pA (Debanne et al. 1995). Therefore these solutions are not considered any further.

### Response to steady dendritic applied current

The bifurcation diagram, using *I*_Dapp_ as the bifurcation parameter (Fig. 4a), is qualitatively very similar to that for *I*_Sapp_, although there are some differences mainly in the curve of steady states. The bottom branch of this curve consists of stable nodes, representing the hyperpolarised resting state (see Fig. 4di, *I*_Dapp_=−1). Stability is again lost via a saddle-node bifurcation (SN1) at *I*_Dapp_=0.02728, leading to a branch of saddles which again turns around at another saddle-node bifurcation (SN2) at *I*_Dapp_=−83.33, before regaining stability again via a supercritical Hopf bifurcation (HB) at *I*_Dapp_=99.78. Notably, *I*_Dapp_ at the HB is much greater than that for *I*_Sapp_, and thus there is a larger range of *I*_Dapp_ for which there are periodic solutions, despite the rheobase being similar. The branch of stable nodes arising from the HB, which represent the depolarised resting state (see Fig. 4di, *I*_Dapp_=120) this time loses stability at a third saddle-node bifurcation (SN3) at *I*_Dapp_=127.6, giving rise to a final branch of saddles.

The branch of stable periodic spiking solutions emanating from the HB again grows in amplitude and period with decreasing *I*_Dapp_ (see Fig. 4dvi, *I*_Dapp_=70) until stability is lost between the two torus bifurcations (TR1 at *I*_Dapp_=15.59 and TR2 at *I*_Dapp_=28.75). Within this unstable branch, there is again a form of waxing and waning spike amplitude (see Fig. 4dv, *I*_Dapp_=25). For decreasing *I*_Dapp_ below that of TR2, a branch of stable, regular periodic spiking solutions appears (see Fig. 4div, *I*_Dapp_=12) before stability is lost again for *I*_Dapp_<9.127 at a period doubling bifurcation (PD). The final unstable branch of periodics for low *I*_Dapp_ disappears at *I*_Dapp_=−3.486 again via a homoclinic bifurcation (HC). Within this unstable branch, the system transitions for decreasing *I*_Dapp_ from doublet activity for *I*_Dapp_ between ≈ 7-9, to a mixture of single spikes and doublets for *I*_Dapp_ of ≈ 6, to then aperiodic activity with a varying number of spikes per burst between *I*_Dapp_ of ≈ 2.6 and 5 (see Fig. 4diii, *I*_Dapp_=4), to finally periodic VLF bursting between HC and *I*_Dapp_ of ≈ 2.5 (see Fig. 4dii, *I*_Dapp_=0.3). Again the HC was confirmed by showing a very high period limit cycle solution (1.77×10^7^) for this value of *I*_Dapp_ (Fig. 4b), which we use as an approximation of the homoclinic orbit, and with which the SN1 point did not coalesce (Fig. 4c).

It is not entirely surprising that the bifurcation diagrams presented in Figs. 3a and 4a are qualitatively very similar, given that the two compartments are coupled quite closely. Nevertheless, there is clearly a larger range of *I*_Dapp_ within which the system is between the hyperpolarised resting and stable spiking states. This suggests that preferentially dendritic depolarisation could engage a wider repertoire of active behaviours and that dendritic currents could be in a more privileged position to shape these behaviours.

### Dependence of model cell behaviour on *g*_Ca_

Indeed lowering the maximal conductance through the dendritically-located, persistent calcium channel (*g*_Ca_) has been shown to switch bursting to spiking activity for a set value of applied current (Pinsky and Rinzel 1994; Traub et al. 1991). A change in *g*_Ca_ from 10 to 7 is sufficient to switch the behaviour of the Pinksy-Rinzel cell from intrinsic bursting to tonic firing with frequency accommodation to better represent the firing properties of CA1 cells (Taxidis et al. 2012). We therefore investigated how *g*_Ca_ affects the bifurcation structure of the system, in an attempt to understand this change mathematically.

**Fig. 5:**
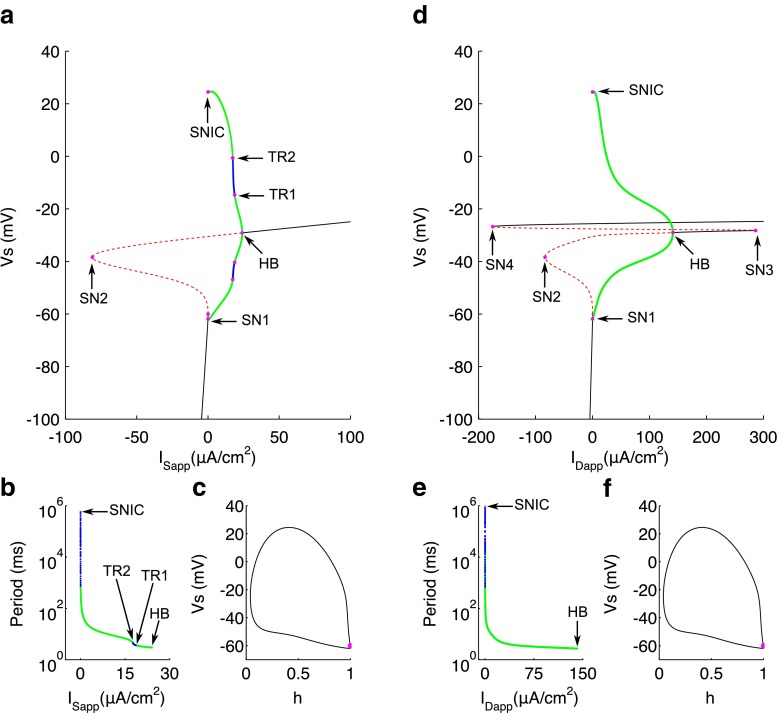
Bifurcation analysis of the CA1 cell for applied somatic and dendritic current. NB. For these diagrams *g*_Ca_=7**a** Projection of the bifurcation diagram with *I*_Sapp_ as the bifurcation parameter. Stable nodes (*black line*), saddles (*red dashed line*), stable periodic orbit maxima and minima (*green dots*), unstable periodic orbit maxima and minima (*blue dots*), bifurcation points (*magenta dots*). SN1 saddle node bifurcation 1 (*I*_Sapp_=0.0557), SN2 saddle node bifurcation 2 (*I*_Sapp_=−81.11), HB supercritical hopf bifurcation (*I*_Sapp_=24.01), TR1 torus bifurcation 1 (*I*_Sapp_=18.73), TR2 torus bifurcation 2 (*I*_Sapp_=17.37), SNIC saddle-node on invariant circle bifurcation (*I*_Sapp_=0.0556). **b** Period of limit cycle solutions for *I*_Sapp_ as the bifurcation parameter. **c** Phase portrait in *V*_*s*_ and *h* at the SNIC bifurcation for *I*_Sapp_. SN1 is represented by a magenta dot. **d** Projection of the bifurcation diagram with *I*_Dapp_ as the bifurcation parameter. SN1 at *I*_Sapp_=0.05745, SN2 at *I*_Dapp_=−83.33, HB at *I*_Dapp_=141.0, SN3 saddle node bifurcation 3 at *I*_Dapp_=288.3, SN4 saddle node bifurcation 4 at *I*_Dapp_=−175.2 and SNIC at *I*_Dapp_=0.0574. **e** Period of limit cycle solutions for *I*_Dapp_ as the bifurcation parameter. **f** Phase portrait in *V*_*s*_ and *h* at the SNIC bifurcation for *I*_Dapp_. SN1 is represented by a magenta dot

We initially re-computed the bifurcation diagrams in *I*_Sapp_ and *I*_Dapp_ for the CA1 value of *g*_Ca_=7 (Fig. 5). Using *I*_Sapp_ as the bifurcation parameter, the bifurcation diagram (Fig. 5a) is extremely similar to that of the CA3 cell with one noticeable difference; instead of the periodic solutions disappearing for low *I*_Sapp_ via a HC bifurcation, they disappear via a saddle-node on an invariant circle (SNIC) bifurcation. The saddle-node bifurcation of equilibrium solutions corresponding to this value of *I*_Sapp_ is that of SN1. This was confirmed by depicting in the *V*_s_−*h* phase plane that the SN1 equilibrium bifurcation lies on the limit cycle at the SNIC bifurcation (Fig. 5c) and that this limit cycle used to approximate the SNIC orbit has high period (5.65×10^5^) (Fig. 5b). In addition, whereas a PD bifurcation was associated with the loss of stability of periodic solutions in the CA3 cell, no such transition was detected for the CA1 cell.

Using *I*_Dapp_ as the bifurcation parameter, there are two noticeable differences between the CA3 and CA1 cell. Firstly while the upper branch of unstable steady states did not bifurcate back to a stable solution in the range of −500≤*I*_Dapp_≥500 for the CA3 cell, in the CA1 cell a fourth saddle-node bifurcation was detected for *I*_Dapp_=−175.2, where the steady state solutions turn around again, giving rise to a second depolarised steady state. This means that in the range of *I*_Dapp_ between SN1 and SN3, the system is bistable (either periodic spiking and depolarised steady states between SN1 and HB, or two depolarised steady states between HB and SN3, depending on the initial parameters). It is noted, however, that a similar behaviour might be observed in the CA3 cell if the bifurcation analysis was extended beyond −500≤*I*_Dapp_≤500 range. Secondly, no torus bifurcations were detected for the periodic spiking solutions in the CA1 cell, whereas a pair of torus bifurcations were present in the CA3 cell. Whilst this may be of mathematical interest, as discussed above, the range of *I*_Dapp_ for which this difference manifests suggests it has no physiological role. As for *I*_Sapp_, the periodic solution disappears for low *I*_Dapp_ via a SNIC bifurcation instead of a HC bifurcation, as was the case for the CA3 cell. Therefore for all *I*_Sapp_/ *I*_Dapp_ between the SNIC at *I*_Sapp_=0.0556, *I*_Dapp_=0.0574 and the HB at *I*_Sapp_=24.01, *I*_Dapp_=141.0 (excluding the regions between the pair of torus bifurcations for *I*_Sapp_), the CA1 cell limit cycle solutions will be periodic spiking with decreasing amplitude and period as *I*_Sapp_/ *I*_Dapp_ increase, with importantly no regimes of bursting or aperiodic and spike doubling behaviours.

**Fig. 6:**
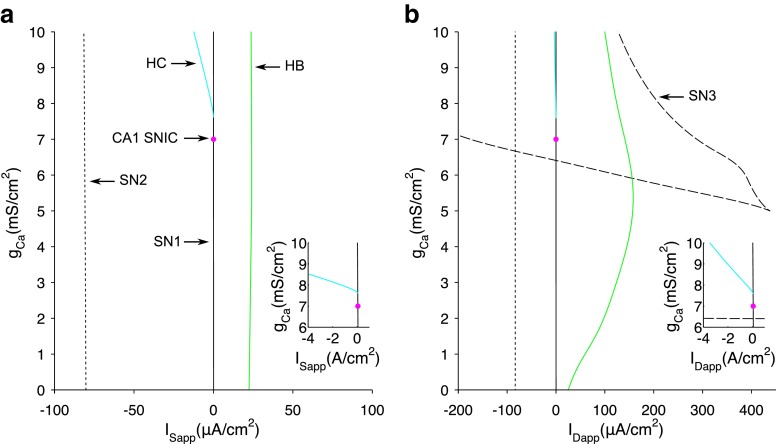
Projection of the 2 parameter continuation in *I*_Sapp_ (**a**) or *I*_Dapp_ (**b**) and *g*_*Ca*_. *Solid black line* - SN1, *dotted black line* - SN2, *dashed black line* - SN3, *green line* - HB, *blue line* - HC, *magenta dot* - SNIC bifurcation for CA1 cell. *Inset* - zoomed in at the codimension-2 SNIC bifurcation where the HC and SN1 curves meet. To construct these diagrams, the bifurcations found in *I* were then continued in the *g*_Ca_ parameter space such that a horizontal slice through these diagrams (e.g. *g*Ca=10) would be the same as the 1-parameter bifurcation diagram in *I* for this value of *g*_Ca_ (i.e. that of Figs. 3–4)

**Fig. 7:**
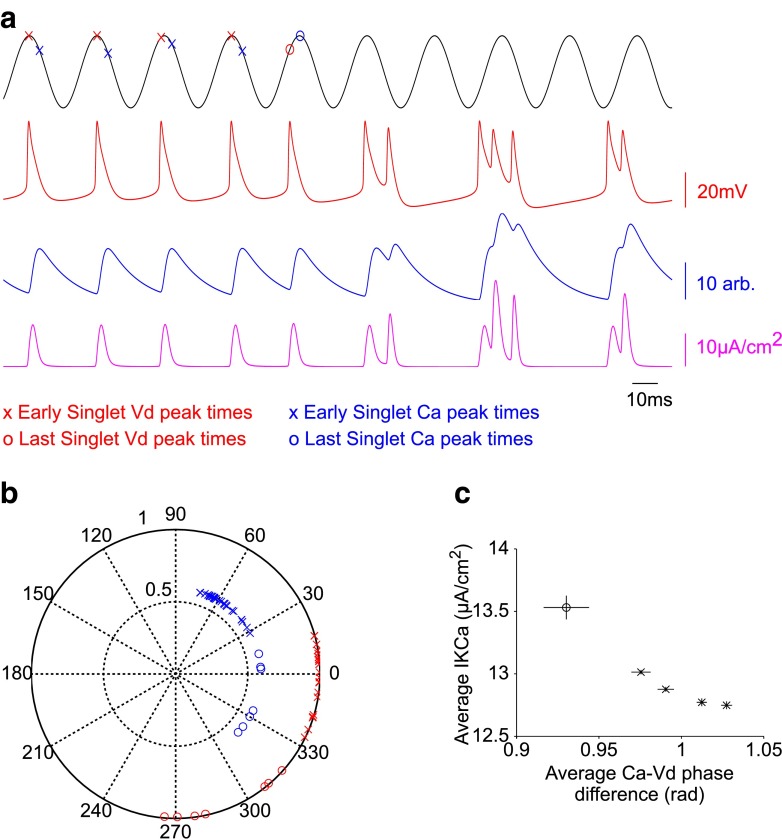
Role of *C* *a* in irregular spiking activity. **a** Example trace of a irregular spiking activity whereby a train of singlets transition into doublet and triplet activity. *V*_d_ (*red*), *C* *a* (*blue*) and $I_{\mathrm {K_{\text {Ca}}}}$ (*magenta*). The oscillation used to calculate phase is shown above (*black*). De-marked atop of the oscillation are the early singlet *V*_d_ (*red x*) and *C* *a* (*blue x*) peak times. The oscillation period was set by the mean inter-spike interval of these early singlet *V*_d_ peaks, and was shifted to minimise the distance between the oscillation peak and the early singlet *V*_d_ peaks. Notice how the last singlet *V*_d_ (*red o*) and *C* *a* (*blue o*) peaks in the singlet train are phase-advanced in the oscillation. **b** Polar plot of the *V*_d_ and *C* *a* peak phases taken from 7 cases where there are at least 5 consecutive singlets before transition to doublet activity. 0 degrees is the peak of the oscillation and 180 is the trough. The radial axis (0−1) shows the normalised *V*_d_ or *C* *a* amplitude (relative to maximum range). The last singlet *V*_d_ and *C* *a* peaks (o) are consistently phase-advanced compared to the early singlet peaks (x). **c** Average *C* *a*−*V*_d_ phase difference against the average peak $\protect I_{\mathrm {K}_{\text {Ca}}}$. There is a fairly consistent *C* *a*−*V*_d_ phase difference and $\protect I_{\mathrm {K}_{\text {Ca}}}$ for the early singlets (x). However for the last singlet in the train (o), the *C* *a*−*V*_d_ phase difference is reduced and this corresponds to a higher $\protect I_{\mathrm {K}_{\text {Ca}}}$

The disappearance of these regimes is likely directly attribute to the presence of a codimension-2 bifurcation in the (*I*, *g*_Ca_) parameter space (Fig. 6a-b), which causes the periodic solution to switch from vanishing via a HC to vanishing via a SNIC, as *g*_*Ca*_ is decreased. The dependence of this regime change on *g*_*Ca*_ also implies that *Ca* plays an important role in shaping both irregular spiking and bursting behaviours. To examine this further, we initially explored how switches between singlet activity and doublet activity during the irregular spiking regime may be shaped by *Ca*, for example when *I*_Sapp_=2. As shown in Fig. 7, during the transition between repetitive singlet activity and doublet activity, the *V*_d_ and *Ca* peaks phase advance but to different extents, leading to a reduction in the (*Ca*−*V*_d_) phase difference and an increase in the peak $I_{\mathrm {K}_{\text {Ca}}}$ which drives the membrane potential to a more hyperpolarised level. This train of events to shift singlet activity to doublet activity seems plausible given changes in $g_{\mathrm {K}_{\text {Ca}}}$, and hence $I_{\mathrm {K}_{\text {Ca}}}$, have previously been shown to shift a cell’s behaviour from spiking to bursting (Tsaneva-Atanasova et al. 2007; Nowacki et al. 2010; Szalai et al. 2011; Iosub et al. 2015).

### Mechanisms of bursting

**Fig. 8:**
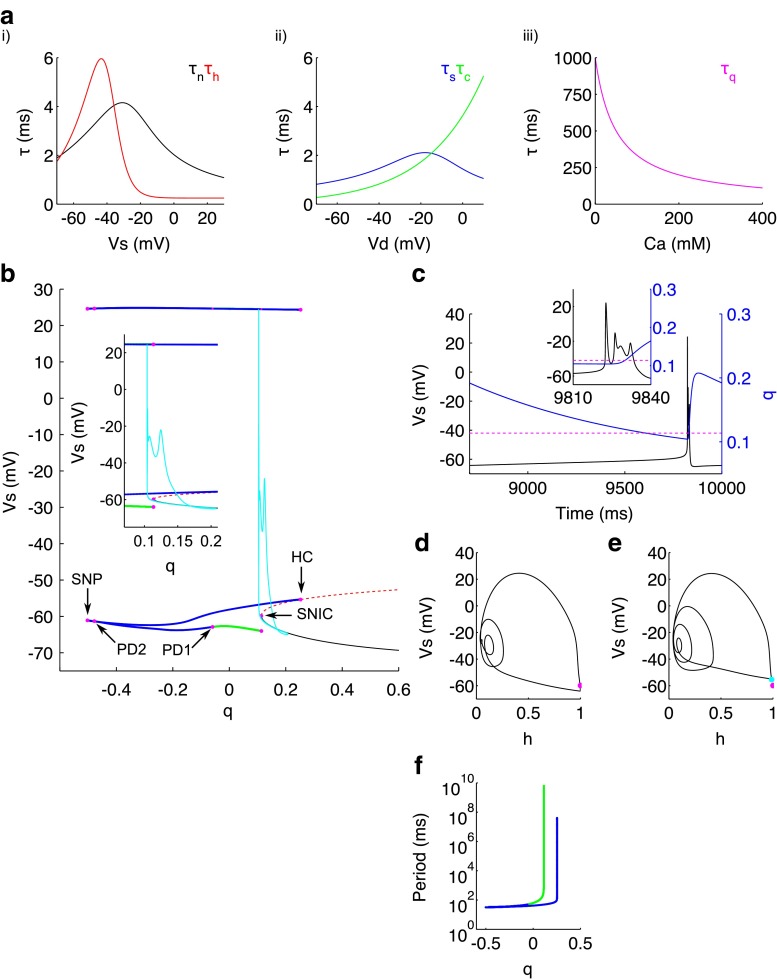
Bifurcation structure in *q* for CA3 bursting. **a** Gating variable time constants within the effective range of *V*_s_ (i), for *τ*_n_ (*black*) and *τ*_h_ (*red*); *V*_d_ (ii) for *τ*_s_ (*blue*) and *τ*_c_ (green); and *C* *a* (iii) for *τ*_q_ (*magenta*). **b** Bifurcation diagram of the fast subsystem with *q* as the bifurcation parameter. Inset - zoomed in around the values of interest for *q*. Stable nodes (*black line*), saddles (*dotted red line*), unstable bursting orbit maxima and minima (*blue lines*), stable bursting orbit (*green lines*), bifurcation points (*magenta dots*), and burst trajecory in the *q*−*V*_s_ plane (*cyan*). SNIC saddle node on invariant cicle bifurcation; PD1-2 period doubling bifurcations 1-2; SNP saddle node bifurcation of periodic solutions; HC homoclinic bifurcation. **c** Time course of the burst for *V*_s_ (*black*) and *q* (*blue*). A dotted red line representing the value of *q* at the SNIC is overlayed. Phase portrait in *V*_s_ and *h* at the SNIC bifurcation (magenta dot) (**d**) and HC bifurcation (**e**) - note the lack of overlap between the SNIC (magenta dot) and the saddle at the HC (*cyan dot*). **f** Period of bursting solutions

Finally we study how *Ca* and the *Ca*-dependent *q* dynamics shape the bursting behaviour in the model. In the original paper, the VLF bursting dynamics of the CA3 cell are described to be largely determined by *q* which, when passes below a threshold, was thought to trigger the initial somatic spike of the burst. As such, bursting was not thought to persist when *q* was replaced by its mean value (Pinsky and Rinzel 1994). Since then, the idea that *q* acts as the threshold for burst initiation has been called into question from phase-plane analysis on a piecewise reduced model (Booth and Bose 2001; Bose and Booth 2005). We therefore performed fast-slow analysis to probe the mathematical mechanism behind the bursting dynamics further. Since both *Ca* and *q* decay on slower timescales (13.33ms and 100−1000ms respectively) than the other gating variables (<6ms within the effective ranges of *V*_s_ and *V*_d_ see Fig. 8a) (Pinsky and Rinzel 1994)) and therefore the ratio of timescales between fast and slow variables is <<1, this analysis is appropriate to investigate how both the *Ca* and *q* dynamics influence bursting. The fast-slow method was originally pioneered by Rinzel (1987), and involves separating the full system into a fast subsystem and a slow subsystem, where the slow variables (in this case *Ca* and *q*) can be used as parameters for bifurcation analysis of the fast subsystem. We analysed the VLF bursting dynamics for both applied somatic current and applied dendritic current. *I*_Sapp_ or *I*_Dapp_ were set to 0.3 with the other equal to 0 (values that generates VLF bursting in the full system). The same qualitative bifurcation structure was observed in both cases. Therefore we present only the bifurcation diagrams for *I*_Sapp_, whilst describing both bifurcation values for the fast subsystem, where *I*_Sapp_=0.3/ *I*_Dapp_=0.3.

We begin our fast-slow analysis using *q* as the bifurcation parameter. For steady applied current, the bifurcation diagram of the fast subsystem (Fig. 8b) is formed of a curve of steady states and a curve of bursting periodic orbits. Since the system continues to burst while *q* is fixed, the intraburst dynamics are independent of *q*, and this variable (*q*) cannot act as a threshold for burst initiation, confirming results in (Booth and Bose 2001; Bose and Booth 2005). To elaborate, the curve of steady states has a lower branch of stable nodes, representing the hyperpolarised resting state, which loses stability for decreasing *q* via a saddle node on invariant circle (SNIC) bifurcation at *q*=0.1136/ *q*=0.1119 (see Fig. 8d) and gives rise to an upper branch of saddles. From the SNIC, a stable branch of bursting periodic solutions also emerges with initially high period (Fig. 8f). As *q* decreases, the amplitude of the burst remains fairly constant while the period decreases. Stability is lost at *q*=−0.05923/ *q*=−0.06627 via a period doubling bifurcation (PD1) then regained briefly via a second period doubling bifurcation (PD2 at *q*=−0.4779/ *q*=−0.4844) before being lost again via a saddle node of periodics (SNP) bifurcation at *q*=−0.5024/ *q*=−0.528. At this point, the bursting periodic orbit branch turns around and increases in period for increasing *q* (Fig. 8f) until it terminates via a homoclinic bifurcation (HC at *q*=0.2524/ *q*=0.2653, see Fig. 8e). Following the burst trajectory (shown in cyan in Fig. 8b), the phase point tightly follows the branch of stable nodes for decreasing *q* and then jumps to the maxima of the bursting trajectory once past the SNIC. In the full system, for this value of applied current, this transition takes several hundred ms (Fig. 8c), again confirming that the threshold for burst initiation is not described by the *q* dynamics. Midway through the burst, *q* then begins to increase which pushes the system back through the SNIC. After this point, the phase point transiently oscillates before returning to the branch of stable nodes. Therefore since the transition below the SNIC in *q* permits the occurrence of the burst, the time between *q* increasing above the SNIC and then decreasing below the SNIC strongly controls at least part of the interburst interval; a finding consistent with previous work (Bose and Booth 2005).

**Fig. 9:**
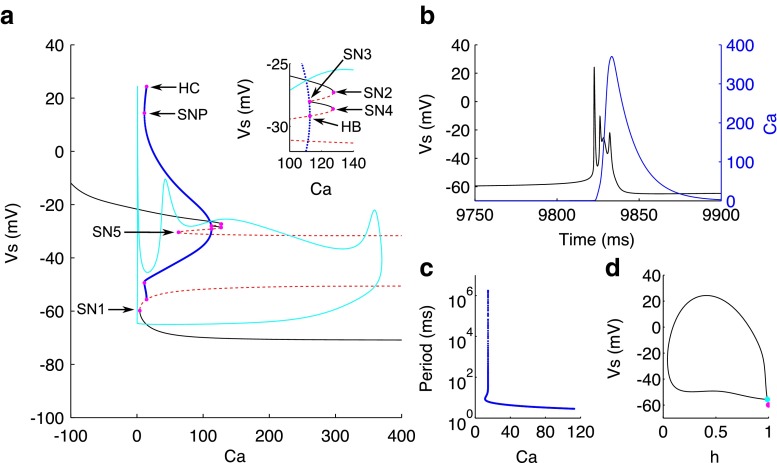
Bifurcation structure in *C* *a* for CA3 bursting. **a** Bifurcation diagram of the fast subsystem with *C* *a* as the bifurcation parameter. Inset - zoomed in around the hopf bifurcation. Stable nodes (*black line*), saddles (*dotted red line*), unstable periodic orbit maxima and minima (*blue line*), bifurcation points (*magenta dots*), and burst trajectory in the *C* *a*−*V*_s_ plane (*cyan*). SN1-5 saddle node bifurcations 1-5; HB subcritical hopf bifurcation; SNP saddle node bifurcation of periodic solutions; HC homoclinic bifurcation. **b** Time course of the burst for *V*_s_ (*black*) and *C* *a* (*blue*). C) Period of limit cycle solutions. **d** Phase portrait in *V*_s_ and *h* at the homoclinic bifurcation point. Note the lack of overlap between SN1 (*magenta dot*) and the saddle at the HC (*cyan dot*)

Given that *q* could not fully describe the intraburst dynamics, we then turned our attention to the second slow variable, *Ca* and performed fast-slow analysis using *Ca* as the bifurcation parameter. For steady applied current, the bifurcation diagram in *Ca* (Fig. 9a) has two long branches of stable nodes, an upper branch representing a depolarised steady state for values of *Ca*<127.5/ *Ca*<127.6 and a lower branch representing a hyperpolarised steady state for *Ca*>4.263/ *Ca*>4.117. Stability in the lower branch is lost for decreasing *Ca* via a saddle node bifurcation (SN1) at *Ca*=4.263/ *Ca*=4.117. The resulting unstable branch of saddles turns around and continues for larger *Ca* values. Stability in the upper branch is also lost via a saddle node bifurcation (SN2) at *Ca*=127.5/ *Ca*=127.6. The branch turns around briefly and is formed of a series of saddles until stability is regained momentarily between another couple of saddle node bifurcations (SN3 at *Ca*=112.5/ *Ca*=112.6 and SN4 at *Ca*=127.2/ *Ca*=127.4). After SN4, the upper branch consists of a second series of saddles which turn around at a fifth saddle-node bifurcation (SN5 at *Ca*=62.76/ *Ca*=63.73) before continuing on to larger values of *Ca*. Between SN4 and SN5, there is a subcritical Hopf bifurcation (HB) at *Ca*=112.7/ *Ca*=113.9 which gives rise to the unstable periodic solutions. For decreasing *Ca*, the amplitude and period of the solution increases (Fig. 9c), before turning around at the saddle node of periodics (SNP at *Ca*=11.21/ *Ca*=11.92) and terminating via a homoclinic bifurcation (HC at *Ca*=14.58/ *Ca*=15.23) (Fig. 9d). Following the burst trajectory, it can be seen that the phase point, loosely follows the lower branch of stable nodes for decreasing *Ca* and overshoots SN5 before jumping to the upper branch of stable nodes. Again the attraction is weak, because the *Ca* dynamics are only intermediately slow, and so the phase point transiently oscillates around this upper branch. Even when the phase point is past the HB, there are smaller oscillations around the unstable upper branch before returning to the attraction of the lower branch of steady states. VLF bursting is therefore governed by the *Ca* dynamics and is of the fold/subHopf type, adoping the nomenclature in Izhikevich (2000), which has been reported also for biophysical models of pituitary somatotrophs and lactotrophs (Stern et al. 2008; Tsaneva-Atanasova et al. 2007; Tabak et al. 2007).

## Discussion

### Mechanisms of spiking

The discontinuous functions in the original Pinsky-Rinzel model were modified to facilitate numerical bifurcation analysis on the full system (Fig. 1), whilst maintaining the qualitative behaviour of the original model (Fig. 2). We show that spiking behaviour emerges in the model from a supercritical Hopf bifurcation and that the rheobase is formed from a saddle-node bifurcation (Figs. 3 and 4). This mechanism of spiking is therefore similar to the bifurcation structure, described earlier, for a single compartmental CA3 model (Grobler et al. 1998). As *g*_Ca_ is decreased, a codimension-two SNIC bifurcation switches the periodic spiking solutions from terminating via a homoclinic bifurcation to terminating via a SNIC bifurcation (Fig. 6). This eliminates the unstable periodic regime, where bursting and irregular spiking solutions exist, and thus explains why a reduction in *g*_Ca_ leads to unperturbed spiking for increasing *I*_Dapp_ and a larger parameter range of *I*_Sapp_ (Fig. 5), as previously observed (Pinsky and Rinzel 1994; Traub et al. 1991). The disappearance of irregular spiking as *g*_Ca_ decreases, is suggestive that *Ca*, as well as shaping bursting behaviour, is also important in shaping spiking in the model. Indeed we show that a reduction in the (*Ca*- *V*_d_) phase difference, and corresponding increase in $I_{\mathrm {K}_{\text {Ca}}}$ is likely to drive activity from singlet spiking to doublet/triplet spiking in this irregular spiking regime (Fig. 7).

As an example of how understanding where Pinsky-Rinzel cells sit dynamically is important for understanding spiking in larger networks, we turn out attention to a combined CA3 and CA1 hippocampal network model, of Pinsky-Rinzel cells that exhibits sharp wave ripple (SWR) oscillations (Taxidis et al. 2012). SWRs are transient network events that occur during periods of awake immobility and slow wave sleep (Buzsaki et al. 1983; Buzsaki 1986; O’Neill et al. 2010), and are known to be important for spatial learning and memory (Girardeau et al. 2009; Ego-Stengel and Wilson 2010; Jadhav et al. 2012). During sharp wave ripples, hippocampal place cells that fire in discrete locations of an environment (O’Keefe and Dostrovsky 1971), reactivate such that co-active place cell activity, representing a discrete environmental location, or sequences of place cell activity, representing a trajectory through an environment, are replayed (Wilson and McNaughton 1994; Nadasdy et al. 1999; Lee and Wilson 2002; Foster and Wilson 2006; Csicsvari et al. 2007; Diba and Buzsaki 2007; Davidson et al. 2009; Karlsson and Frank 2009). However the mechanisms that select which place cells are active in SWRs is still unclear, although the intrinsic excitability of the cells and the local synaptic connectivity structure of the network within which they reside are both likely to play a role (for review see Atherton et al. (2015)).

In the Taxidis model (Taxidis et al. 2012), CA1 cells are driven to spike by CA3 cell activity. From a dynamical systems perspective, the occurrence of a single CA1 spike could be influenced by CA3 in two ways. Firstly, if the CA1 cell was in a spiking limit cycle regime, excitatory input from CA3 could phase shift CA1 cell spike times. Secondly, if the CA1 cell was in a hyperpolarised resting state, excitatory input from CA3 could push the CA1 cell trajectory through the phase space in a manner that resembles a spike, but which does not actually push the CA1 cell trajectory onto a stable limit cycle i.e. causes a transient. The bifurcation analysis, conducted here, facilitates the differentiation between these quite different mechanisms of CA1 spiking, namely periodic attractor driven verses transient oscillations. The analysis shows that for the parameter values of *I*_Sapp_ and *g*_Ca_ used in the model (0 and 7 respectively), the CA1 cells are in a hyperpolarised resting regime below the SNIC. Therefore at least in this model, it can be concluded that CA1 cell spiking in SWRs is driven by transient oscillations, induced by presynaptic CA3 cell activity, and not by a periodic attractor driven excitability.

### Mechanisms of bursting

Experimental findings have shown that the intrinsic bursting of CA3 cells is driven by several processes: the intital fast spike is driven by activity through sodium channels; the ensuing after depolarising potential (ADP) subsequently drives the burst; and the burst is then terminated by an after hyperpolarising potential (AHP) (Wong and Prince 1978; 1981). Although the ionic bases for the ADP and AHP that occur during bursting in CA3 pyramidal cells are not fully understood, they are both calcium driven (Wong and Prince 1978; 1981; Schwartzkroin and Stafstrom 1980) and are likely mediated by T/R-type calcium channels and calcium-activated potassium channels respectively, as has been found in some cases for CA1 cell bursting (Magee and Carruth 1999; Metz et al. 2005; Alger and Nicoll 1980), although see (Azouz et al. 1996). Kv7/KCNQ/M-channels also play a role in terminating the ADP (Vervaeke et al. 2006; Brown and Randall 2009).

Previous studies using fast-slow analysis of CA3 pyramidal cell models have proposed different mechanisms as underlying bursting activity (Kepecs and Wang 2000; Xu and Clancy 2008). In a reduced version of the Pinsky-Rinzel model with similar somatic currents but only a persistent sodium current and a slow potassium current in the dendritic compartment; bursting activity was found to be of the square-wave/fold-homoclinic type and dependent on the activation variable of the slow potassium current (Kepecs and Wang 2000). In contrast, using fast-slow analysis on a single-compartmental CA3 model, incorporating various additional currents to the Pinsky-Rinzel model, bursting was found to be of the parabolic type and dependent on two variables: the slow autocatalytic activation variable for a T-type calcium channel and the activation variable for the slow calcium-dependent potassium current (Xu and Clancy 2008).

It remained to be established which, if either, of these mechanisms is true for the original Pinsky-Rinzel model, where the two candidate slow variables for controlling bursting are calcium and the activation gate, *q*, of the dendritic after hyperpolarisation current. Here, we identify a mechanism intermediate between the two cases, whereby bursting is of the fold/sub-Hopf type and relies on just one variable, calcium, which is dependent on the dendritic calcium current, *I*_Ca_, and in fact controls both the calcium-dependent potassium current, $I_{\mathrm {K}_{\text {Ca}}}$, and the activation gate, *q*, of the dendritic after hyperpolarisation current, $I_{\mathrm {K}_{\text {AHP}}}$. The rise of *Ca* through the subcritical Hopf bifurcation mediates the slow termination of the burst (Fig. 9), thereby biophysically explaining previous results on a piecewise reduced system, showing disruption of the ping-pong mechanism and termination of the burst as *I*_Ca_ was activated (Booth and Bose 2001; Bose and Booth 2005). The results, here, are also consistent with the blockade of calcium channels perturbing CA3 bursting Wong and Prince (1978, 1981) which could not be explained by bursting dependent on activation of a slow potassium current alone (Kepecs and Wang 2000).

The original Pinsky-Rinzel paper proposed that *q* sets the burst initiation threshold (Pinsky and Rinzel 1994). Subsequent work, using the piecewise reduced system, showed this threshold was independent of *q* and instead set by *V*_d_ (Booth and Bose 2001; Bose and Booth 2005). We clarify this point here by showing, using fast-slow analysis, that while *q* plays no role in setting the threshold for the initial somatic spike in the burst or indeed the intraburst dynamics, it does set part of the interburst interval (Fig. 8). The biophysical implications of this is that the dendritic afterhyperpolarisation current plays an important role in setting the interburst interval.

Nevertheless, it it important to note that the dependence of bursting on just one slow variable is likely due to the bistability of the fast subsystem in the Pinsky-Rinzel model which is driven by the window *I*_Na_ (Golomb et al. 2006). This derives from the overlap in the steady-state activation and inactivation curves for the *I*_Na_ current, where the inactivation curve is shifted to more depolarised values (>20*mV*) compared to experimental results (Sah et al. 1988). When this window current was reduced, bursting in a different CA3 model was instead dependent on two slow variables and was of the parabolic type (Xu and Clancy 2008).

## Conclusion

By proposing a modified smooth system of differential equations that can be used to further analyse the full Pinsky-Rinzel model, we hope that future studies using Pinsky-Rinzel cells as part of a network, or as a means to analyse firing patterns will have a tool with which to systematically identify parameter regimes of interest to their research question. Characterising the bifurcation structure for applied current, calcium conductance and applying fast-slow analysis to investigate the bursting regime in the systems already provides insight into the parameter dependence of the model dynamics.
